# Surgical approach to posterior mediastinal Castleman´s disease: a case report

**DOI:** 10.11604/pamj.2025.50.54.45984

**Published:** 2025-02-18

**Authors:** Badreddine Belayachi, Hicham Fenane, Yassine Msougar

**Affiliations:** 1 Department of Thoracic Surgery, CHU Mohammed VI, Marrakesh, Morocco,; 2 Cadi Ayyad University, Marrakesh, Morocco

**Keywords:** Castleman disease, mediastinal neoplasms, thoracotomy, COVID-19, case report

## Abstract

Castleman´s disease (CD) is a rare lymphoproliferative disorder often presenting as a hypervascular mass. This case highlights the unique challenges of surgically managing a posterior mediastinal CD mass adherent to vital structures. A 58-year-old woman was incidentally diagnosed with an asymptomatic posterior mediastinal mass during routine imaging for COVID-19. Computed tomography angiography revealed a 34 x 26 mm hypervascular mass closely associated with the esophagus, pulmonary artery, and bronchus. Initial surgical resection via VATS was converted to a posterolateral thoracotomy due to significant bleeding and adhesions. Histopathological examination confirmed hyaline vascular Castleman´s disease. The patient experienced an uneventful recovery and demonstrated a one-year remission. This case underscores the importance of advanced imaging and intraoperative flexibility in managing rare mediastinal masses. It also highlights the excellent prognosis achievable with complete resection, even in anatomically challenging cases.

## Introduction

Castleman´s disease (CD), also known as “angiofollicular lymph node hyperplasia”, is a rare lymphoproliferative disorder characterized by benign lymph node enlargement with an unclear etiology [1]. The disease can present as unicentric Castleman´s disease (UCD) or a multicentric variant, with UCD often managed surgically. While posterolateral thoracotomy has traditionally been the approach of choice for resection, advancements in minimally invasive techniques, such as video-assisted thoracoscopic surgery (VATS), have increasingly enabled the management of challenging cases involving hypervascular tumours [2-5].

We report a rare case of a posterior mediastinal Castleman´s disease tumour adherent to vital structures, illustrating the complexities of surgical resection and the significance of histopathological diagnosis.

## Patient and observation

**Patient information:** a 58-year-old female with hypertension controlled on amlodipine presented with an incidental posterior mediastinal mass discovered during COVID-19 screening computed tomography (CT) angiography. The patient was largely asymptomatic, with only mild, recurrent chest pain and several café-au-lait spots on her abdomen. No significant weight loss, fatigue, or dysphagia were reported, and routine blood tests were within normal limits.

**Figure 1:**
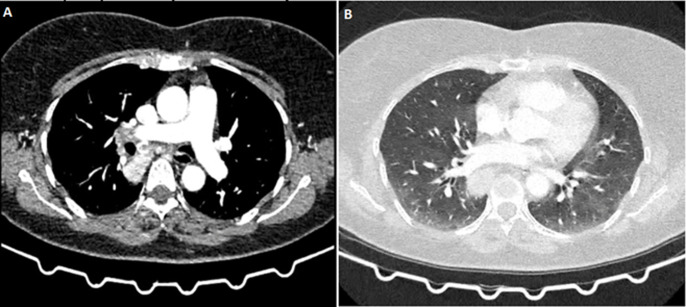
chest CT imaging of a posterior mediastinal mass: A) mediastinal window, highlighting the mass, while panel; B) parenchymal window for better visualization of the surrounding lung tissue

**Clinical findings:** computed tomography angiography revealed a 34 x 26 mm hypervascular, well-circumscribed posterior mediastinal mass with intense contrast enhancement (Figure 1). The mass was closely associated with critical structures including the right pulmonary artery, mainstem bronchus, inferior pulmonary vein, and esophagus, with blood supply from prominent bronchial and dorsal arteries, complicating potential surgical intervention.

**Timeline of the current episode:** the mass was initially identified incidentally during COVID-19 screening, followed by surgical planning and attempted VATS resection, which was converted to posterolateral thoracotomy due to intraoperative bleeding and adhesions, ultimately achieving successful removal of the mass.

**Diagnostic assessment:** the diagnosis relied on radiological findings and subsequent histopathological examination post-surgery, with CT angiography showing a hypervascular mass adherent to vital structures. While magnetic resonance imaging (MRI) and positron emission tomography (PET) scans were not performed, the mass's radiological features and intraoperative findings suggested Castleman's disease.

**Diagnosis:** the mass was definitively diagnosed as unicentric Castleman's disease of the hyaline vascular type, confirmed by histopathological examination post-surgery.

**Figure 2:**
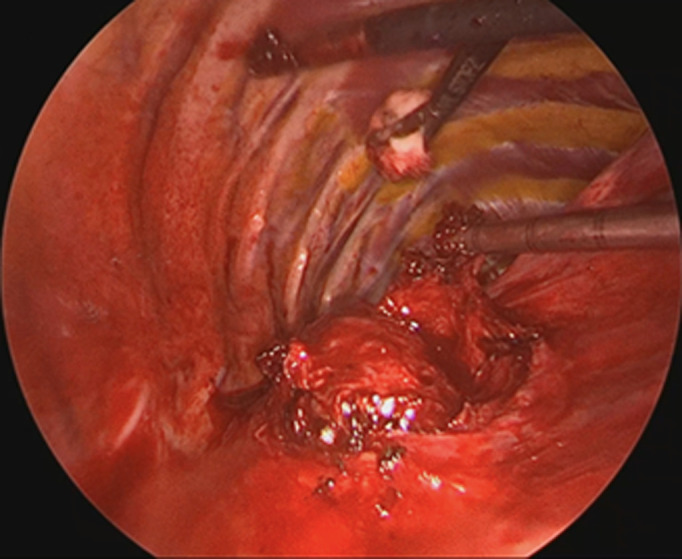
thoracoscopic view of the posterior mediastinal mass with adhesions

**Therapeutic interventions:** initial surgical resection was attempted via VATS, but intraoperative complications-significant bleeding and adhesions-necessitated conversion to a posterolateral thoracotomy. A thorough resection was achieved with a total blood loss of approximately 400 ml (Figure 2).

**Follow-up and outcome of interventions:** postoperatively, the patient recovered without complications. Histopathological analysis confirmed the diagnosis of hyaline vascular Castleman´s disease. The patient was followed up for one year, during which she remained asymptomatic with no recurrence or complications.

**Patient perspective:** the patient reported satisfaction with the treatment, noting the swift recovery post-surgery. She was relieved that the mass, initially found incidentally, was successfully treated and that she remained free from recurrence during the follow-up period.

**Informed consent:** this was obtained from the patient for the publication of this case report and accompanying images.

## Discussion

**Epidemiology and diagnosis:** Castleman´s disease (CD) is a rare lymphoproliferative disorder with an unclear etiology, first described by Castleman and Towne in 1954 [6]. It predominantly affects the thoracic region, though it can also present in areas such as the retroperitoneum, muscles, and cervical regions. Unicentric CD (UCD) typically manifests as compressive symptoms or is discovered incidentally, as in our case [2]. Given its rarity, CD should always be considered in the differential diagnosis of mediastinal masses. Diagnostic imaging, particularly chest CT, is essential in identifying CD, revealing a well-circumscribed, hypervascular mass with intense contrast enhancement. While MRI and PET scans provide additional details regarding the tumor´s extent and metabolic activity, these were not performed in our case [6-10]. The imaging features, including the hypervascular nature of the mass, are crucial for guiding preoperative planning.

**Management:** the treatment of choice for UCD is complete surgical resection, which not only offers a definitive tissue-based diagnosis but also serves as a potential curative intervention. With advancements in VATS technology, more complex cases previously requiring open thoracotomy can now be managed with minimally invasive approaches. VATS has been successfully employed in a growing number of thoracic surgeries, reflecting significant improvements in surgical instruments and techniques. However, as seen in our case, intraoperative challenges, including significant bleeding and adhesions, may necessitate a conversion to thoracotomy. These complications are common in CD surgeries [4,5], emphasizing the need for surgical flexibility. Our patient´s successful recovery highlights the critical role of adapting the surgical strategy to the intraoperative findings, ensuring optimal outcomes.

**Outcomes and non-surgical management:** for patients with UCD, the prognosis following complete resection is generally excellent, with long-term survival rates exceeding 90%. Our patient´s recovery and remission at one-year follow-up support this. Non-surgical treatments, such as radiotherapy, chemotherapy, rituximab, and anti-IL-6 therapy, are reserved for unresectable cases or multicentric CD. While these therapies can offer symptomatic relief and disease control, the limited data on their efficacy highlights the importance of surgical management in UCD [6]. In this context, the absence of postoperative complications and the patient's asymptomatic follow-up further reinforce the benefit of complete resection for achieving long-term remission.

**Discussion and future directions:** the complexity of CD management stems from both its rare presentation and the challenges encountered during surgical intervention. This case adds to the literature by demonstrating the successful application of VATS in managing a complex mediastinal CD case, despite the need for conversion to open thoracotomy. Furthermore, it underscores the importance of accurate preoperative imaging and flexible surgical approaches to address intraoperative complications. However, the lack of advanced imaging, such as MRI or PET scans, limited the preoperative assessment of the tumor´s extent and metabolic activity, which could have influenced surgical planning. Additionally, while the patient´s one-year follow-up was uneventful, longer-term follow-up is necessary to assess recurrence or late complications. Future research should focus on improving minimally invasive surgical techniques and investigating adjunctive therapies to further enhance the outcomes for patients with CD.

## Conclusion

This case highlights the complexities of diagnosing and treating Castleman´s disease, with the posterior mediastinal mass incidentally discovered during a COVID-19 workup. After surgical resection, the mass was identified as hyaline vascular type Castleman´s disease. Although rare, the condition presents a range of challenges, from incidental findings to symptomatic compression. Advanced imaging, particularly chest CT, plays a crucial role in diagnosis and surgical planning. Despite intraoperative complications like bleeding and adhesions, complete resection remains the gold standard, providing a favorable prognosis. Non-surgical therapies are reserved for unresectable cases. This case underscores the importance of surgical flexibility and highlights the need for ongoing research to refine diagnostic and therapeutic strategies for improved patient outcomes.
